# Early Visual Attention Abilities and Audiovisual Speech Processing in 5–7 Month-Old Down Syndrome and Typically Developing Infants

**DOI:** 10.3390/brainsci11070939

**Published:** 2021-07-16

**Authors:** Jovana Pejovic, Marisa Cruz, Cátia Severino, Sónia Frota

**Affiliations:** Center of Linguistics, Lisbon Baby Lab, University of Lisbon, 1600-214 Lisbon, Portugal; marisac@edu.ulisboa.pt (M.C.); catiaseverino@campus.ul.pt (C.S.); sfrota@edu.ulisboa.pt (S.F.)

**Keywords:** attention, audiovisual processing, Down syndrome, communicative abilities in infants

## Abstract

Communicative abilities in infants with Down syndrome (DS) are delayed in comparison to typically developing (TD) infants, possibly affecting language development in DS. Little is known about what abilities might underlie poor communication and language skills in DS, such as visual attention and audiovisual speech processing. This study compares DS and TD infants between 5–7 months of age in a visual orientation task, and an audiovisual speech processing task, which assessed infants’ looking pattern to communicative cues (i.e., face, eyes, mouth, and waving arm). Concurrent communicative abilities were also assessed via the CSBS-DP checklist. We observed that DS infants orient their visual attention slower than TD infants. Both groups attended more to the eyes than the mouth, and more to the face than the waving arm. However, DS infants attended less to the eyes than the background, and equally to the face and the background, suggesting their difficulty to assess linguistically relevant cues. Finally, communicative skills were related to attention to the eyes in TD, but not in DS infants. Our study showed that early attentional and audiovisual abilities are impaired in DS infants, and might underlie their communication skills, suggesting that early interventions in this population should emphasize those skills.

## 1. Introduction

Down syndrome (DS) is associated to a genetic perturbation known as trisomy 21 affecting physical, motor, and cognitive functioning. It is the most common genetic cause of intellectual disability. DS vastly affects language processing and development [1,2,3,4]. Both language comprehension and production deficits have been described. In particular, growth slopes in comprehension become shallower with age and language production studies demonstrate either delayed or atypical speech patterns (for a review, see [5]), especially in childhood and adolescence (e.g., [6]). Speech production in infants and toddlers revealed mixed results (e.g., [7,8]). Phonological acquisition has been reported to be delayed, showing deviant patterns [9], and comprehension and production of prosody has been shown to be impaired in children with DS and adolescents [10]. Equally important, hearing in individuals with DS is often impaired consequently affecting their language learning (for a review, see [3,11]). Language learning difficulties are evident in the late occurrence of first words/signs which appear between 24–36 months of age, while in typically developing children they usually occur between 12 and 18 months of age. In addition to language processing, DS 26-month-old toddlers show impairment in their social communication abilities (e.g., [12]). Taken together, speech impairment in DS extends to later developmental stages affecting DS individuals’ overall communicative skills, and possibly academic success, and general well-being.

Many studies identified the benefit of early parent-implemented intervention in DS children younger than three years of age for their further language skills (for a review, see, e.g., [12]). One of the main aims of these early interventions in DS children is to target abilities that might relate to later language outcomes. For instance, a recent meta-study on joint attention demonstrated that this ability is rather a strength than a weakness in DS population [13]. Developmentally, joint attention refers to a nonverbal skill occurring in social interaction between an infant and a caregiver. Using eye-gaze cues, pointing gestures or vocalizations, attention between the infant and the caregiver is focused/shared to the same object/event, and accompanied by awareness that the attentional focus is shared (e.g., [14]). Joint attention in typically developing populations is related to object learning [15], word learning (e.g., [16,17]; but see other proposals [18,19]), or later language outcomes (e.g., [20]). Similarly, joint attention is relevant for word learning in Down syndrome children as well [21], and it is a strong predictor in DS infants for later expressive and receptive language outcomes [12]. However, this skill emerges chronologically later in DS children (in their second year of life) than in typically developing children (for a review, see, e.g., [22]), suggesting that precursors to joint attention development might be impaired in Down syndrome children. In the present study, we will focus on some of these possible cognitive abilities that might directly or indirectly support further language development in DS population. 

To support initial communicative abilities, infants have to learn to take part in non-verbal communication (i.e., joint attention), but they also have to selectively attend to relevant social communicative cues. In particular, they need to attend to faces and communicative gestures, and to process visual communicative cues accompanying the auditory speech signal (i.e., articulatory movements, eyebrows and head movements, gestures, etc.). In adults, attending to these cues facilitates face-to-face communication in noisy conditions (e.g., [23,24]). In infants, visual cues may support phonetic and word learning [25,26], as well as the learning of syntax [27]. Importantly, visual cues are available to infants already in early infancy—by four months of age infants are able to integrate auditory and visual information (e.g., [28,29,30]). Thus, the ability to attend to visual communicative cues develops early in infancy and is important for language development. Understanding infants’ attention to visual communicative cues in atypically developing populations is particularly relevant, especially for infants undergoing speech interventions that are often based on improving communicative abilities. 

Studies investigating the ability to process visual communicative cues suggest that DS infants are delayed in comparison to chronologically matched TD infants. For instance, DS infants discriminate between objects and human faces by four months of age, while TD infants do so already by two months of age [31], suggesting impaired ability to detect relevant social communicative cues in DS early development. Further, in a longitudinal study during the first six months of life, TD infants demonstrate a first peak in forming eye contact with their mothers already at one and a halfmonths of age, while DS infants do so around their third month of age [32]. Interestingly, the same study revealed that once DS infants form eye contact, they maintain it longer than the TD group, possibly affecting their ability to shift their gaze towards other objects in their environment that a caregiver is gazing to. A recent study demonstrated that unlike TD toddlers, DS toddlers at 16 months of age (chronologically age matched with a TD group), and at 28 months (mental age matched with the 16-month-old TD group) are not able to detect a mismatch in the audiovisual speech signal [33]. 

Importantly, attentional (cognitive) impairments in DS infants go beyond the abovementioned impairments in visual speech processing and attention to faces. DS toddlers are slower in disengaging their visual attention from an object they have been engaged to, in comparison to chronologically or mentally-age matched TD infants, as shown by [34]. The same study showed that being faster in visual attention disengagement relates with higher expressive and receptive vocabulary abilities in both TD and DS toddlers. In another study, five-year-old DS children were faster in disengaging than TD children, but similar in how fast they orient (attend) to visual stimuli [35]. Other study yet reported lower performance in DS children from three-six years of age in visual sustained attention [36]. Therefore, results converge in suggesting that DS children and toddlers’ visual attention abilities are impaired in comparison to their TD peers. However, little is known on early visual attention abilities in DS infants, particularly in their first six months of life. Understanding visual attention skills in DS in the first months of life is crucial to understand their reported impairments in early face processing and audiovisual speech processing (e.g., [31,33]), that possibly underlie their impaired language development. 

The current study assessed five-to-seven-month-old DS infants and compared them to a chronologically matched TD group in three separate measures of visual attention, audiovisual speech processing, and communication abilities. Our main goal was to establish what the early relations between the three components are and compare them across the DS and TD groups. We hypothesized that DS infants’ performance in all the three measures would differ from TD infants’ performance. Specifically, we expected that DS infants would show an impairment in visual attention, reflected in slower visual orientation latency, while for the audiovisual task the DS group would attend less to communicative cues than their TD peers. Finally, we expected that the DS group would underperform on measures of communicative abilities in comparison to the TD group. 

## 2. Materials and Methods

### 2.1. Participants

Seven infants with Down syndrome (mean age = 6 months; age range from 5 months and 3 days to 7 months and 20 days; 3 males) and 24 typically developing infants (mean age = 5.25 months; age range from 5 months and 2 days to 6 months and 28 days; 16 males) took part in this study. DS infants were recruited from the Center for Child Development Diferenças in Lisbon, Portugal. They were born full-term and had normal hearing to mild hearing loss and normal or corrected-to-normal vision (according to clinical screening). TD infants were all born full-term, with no reported medical/developmental concerns. Additionally, questionnaires on language and overall development (see details in Materials and Procedure) served as a screening tool to confirm TD infants’ development. All infants were raised in monolingual European Portuguese homes. The study was approved by the Ethical Committee for Research of the School of Arts and Humanities of the University of Lisbon.

### 2.2. Materials and Procedure

Infants took part in two tasks: (1) the visual attention task, and (2) the audiovisual task. First, infants were tested in the visual attention task, followed by the audiovisual task. After completing both tasks, parents provided information on demographic and health status of the infant. Overall communicative development was assessed with the Communicative and Symbolic Behavior Scales Developmental Profile (CSBS-DP) adapted for Portuguese, that measures infants and toddlers’ development from 6 to 24 months of age [37]. The CSBS-DP provides data on several scales: emotion and use of eye gaze, use of communication, use of gestures, use of sounds, use of words, understanding of words, and use of objects. 

#### 2.2.1. Visual Orientation Attention Task

Similar to a previous study on DS and TD children [35], we tested infants in a visual orientation attention task. In this task we measured infants’ looking latency to visual stimuli, here flashing lights. Infants were seated on their caregiver’s lap in a testing booth facing the central green light, while two red lights were placed laterally from the infant. Infants’ looking behavior was monitored on a camera (Logitech c920, Logitech, Fremont, CA, USA) and online coded by an experimenter placed outside the booth. Every trial began by flashing the central green light. Once the infant orients toward it, this light turns off and one of the lateral red lights starts to flash. When the infant directs its look toward the lateral red light, the experimenter records this by pushing a button on the keyboard. The lateral red light continues to flash for 2 s and then turns off, while the green-central light starts flashing and a new trial begins. Infants’ orientation latency is measured as the time between the onset of flashing of the lateral red light and the moment when infants look away from the central green light towards the lateral red light. If an infant did not direct its look to the central light, the experimenter played a short infant-friendly sound to recover infants’ attention to the task. Moreover, if the experimenter noticed that the infant is not attending to the lateral light for a significantly long period (i.e., longer than 8 s), the trial ended by turning off the red light and initiating the green light. The 8-s reference was used as it was the maximum trial duration in [35]. There was a maximum of 10 trials (5 on the left and 5 on the right), and the presentation of the left vs. right lateral trials was randomized. The task stopped if an infant lost interest in the task, therefore the number of trials might vary across infants. Stimuli presentation was controlled by the Look software [38]. The time of infants’ orientation latency from the central green light to the lateral red light was also recorded by the software. 

#### 2.2.2. Audiovisual Task

**Figure 1:**
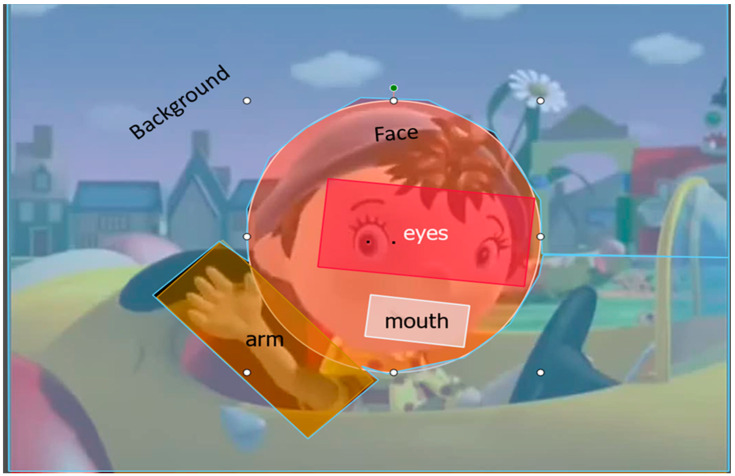
Still-example frame from the audiovisual task. Marked in colors are the areas of interest analyzed in the task: the face, the eyes, the mouth, the arm, and the background.

Infants’ eye-gaze was recorded while watching 4-s-long videos of an animated character (Noddy) talking and waving at the infant (Figure 1). Videos were part of a stress perception task where they were inserted after each block as a reinforcer [39]. Auditorily, four different reinforcing passages were paired with the same video (e.g., “That’s it! We are going to play one more time” (Four following passages were used: “É isso! Vamos jogar mais uma vez” (That’s it! We are going to play one more time); “Muito bem. Vamos continuar o nosso jogo” (Well done! We are going to continue our game); “Muito bem! Este jogo é muito divertido” (Well done! This game is a lot of fun); “Parabéns! Vejo que estás mesmo a gostar disto” (Good! You are really enjoying the game)). The video did not change visually throughout the task, only the auditory passages. The order of presentation of the video with the different passages was randomized between infants. Intentionally, the Noddy character was presented centrally in the video, against a colorful and attractive background, to assess infants’ attention to visual linguistic and paralinguistic communicative cues (the face, the arm) versus non-linguistic objects (the background). In total, infants could be presented with up to eight videos. However, the stress perception task stopped when infants lost interest in the task, therefore infants varied in how many videos they were presented with.

Infants were seated in the caregiver’s lap in a dimmed testing booth, facing the stimuli presentation monitor (Dell LCD screen in 1680 × 1050 pixel resolution) on ~70 cm distance from the monitor. Auditory stimuli were played over speakers (Genious) placed behind the monitor. Infants’ eye-gaze was recorded using the SMI RED500 eye-tracker, whereas the SMI Experimenter Center and iView X software-controlled stimuli presentation.

#### 2.2.3. Overall and Communicative Development Assessment

Parents filled in the CSBS-DP checklist at the time of the audiovisual and attention tasks, since this tool was also used as a screening tool to make sure that the TD group indeed followed a typical development. Not all infants that participated in the attention and audiovisual tasks provided data for the questionnaire, and thus the sample of infants for overall and communicative development assessment differed from that of the audiovisual/attention tasks (see details in the result section). Data from the CSBS-DP were examined through correlation analyses with performance on the audiovisual task.

## 3. Results

### 3.1. Visual Orientation Task

**Figure 2:**
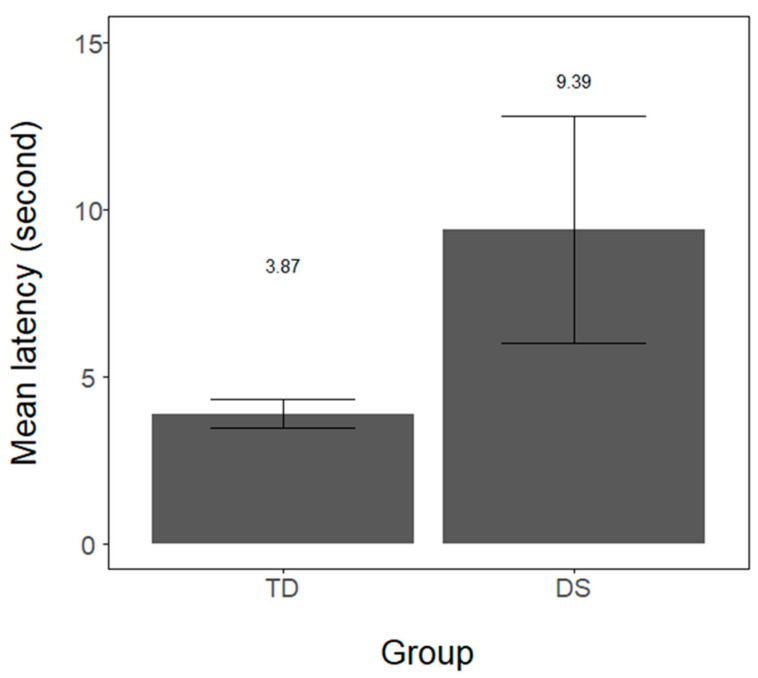
Mean orientation latency (in seconds) in the visual attention task across the Down syndrome (DS) and typically developing (TD) groups. The values above the bars refer to the mean latency value for each group. Error bars represent 1 (+/−) standard error of mean.

Infants’ latency (in seconds) in orienting to the red lateral light was measured for every trial and averaged for each infant. In both groups, the majority of infants completed all 10 trials. In the DS group, 6 out of 7 infants completed 10 trials (M = 9.28, range from 5–10). In the TD sample, 16 out of 24 infants provided data for all trials (M = 8.95; range from 5–10 trials). Infants’ orientation latency for the two groups is provided in Figure 2. Because sample size differed across groups, we performed a linear-mixed model analysis with infants’ orientation latency as the dependent variable, group (DS and TD) as a fixed effect, while by-subject intercept was set as a random effect. Using the lmerTest [40] package in R, we observed that the DS group revealed significantly longer latency than the TD group (intercept = 6.29, DS estimate = 2.46, SE = 0.86, t = 2.85, *p* = 0.008, 95% CI: 0.77–4.16). Additionally, we compared the number of trials longer than 8 s across groups. A Wilcoxon-Mann-Whitney test revealed that DS infants exhibited more trials longer than 8 s (M = 3.0, SD = 2.0) than TD infants (M = 1.29, SD = 1.5; Z = 2.19, *p* = 0.028, r = 0.39).

### 3.2. Audiovisual Task

**Figure 3:**
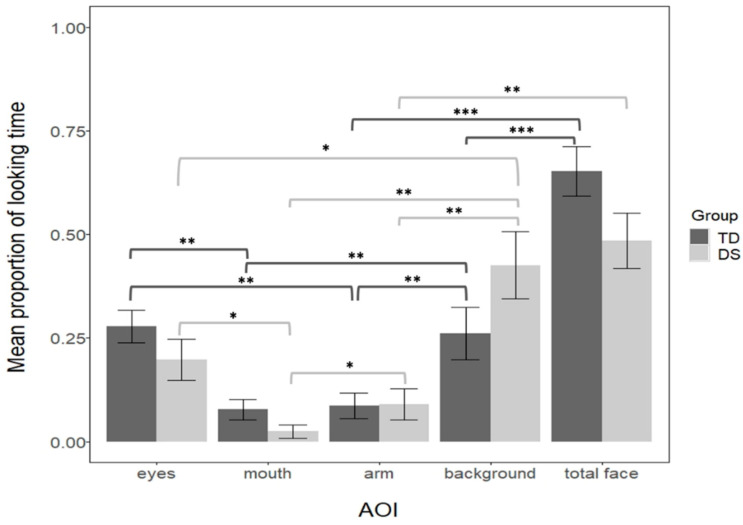
Mean proportion of looking time in the audiovisual task across the Down syndrome (DS) and typically developing (TD) groups. Error bars represent 1 (+/−) standard error of mean. Significant differences are signaled: 0.05 = *, 0.01 = **, 0.001 = ***.

Infants’ looking times to the screen were recorded with an eye-tracker. We defined dynamic areas of interest (AOI) covering the background, the arm, the face, the eyes, and the mouth (Figure 1). For each trial, we calculated the proportion of looking time to the AOIs in comparison to the whole screen. Next, for each infant we averaged proportions across all trials. The total number of trials differed across infants, depending on how long they were interested in the task (between 1 and 8 blocks). A Wilcoxon-Mann-Whitney test revealed that groups did not differ in the number of completed trials (M_TD_ = 3.91, range 2–6; M_DS_ = 3.4, range 2–5; Z = −0.71, *p* = 0.47, r = 0.13). The looking patterns for the two groups of infants are depicted in Figure 3.

The looking pattern was analyzed for each group separately. First, we compared the 4 levels of AOIs (the eyes, the mouth, the arm, and the background) separately for each group. The results revealed that AOIs significantly differed in both groups (a Kruskal-Wallis test for the DS, H(3) = 17.7, *p* = 0.004, η^2^ = 0.61; a one-way-ANOVA for the TD F(3, 92) = 8.82, *p* < 0.001, η^2^ = 0.22). Pairwise comparisons (Bonferroni controlled) showed that regarding the background, both groups looked longer at the background than the arm (both *p*s = 0.001, d_DS_ = 1.3, d_TD_ = 0.35), and longer to the background than the mouth (*p*_TD_ = 0.0012, d_TD_ = 1.8, *p*_DS_ = 0.0017, d_DS_ = 0.66). However, DS infants looked longer at the background than the eyes (*p* = 0.04, d = 0.78), but not TD infants (*p* = 0.86, d = 0.03). Regarding the arm, TD, but not DS infants, looked more at the eyes than the arm (*p*_TD_ = 0.001, d_TD_ = 0.38; *p*_DS_ = 0.2, d_DS_ = 0.47), whereas DS, but not the TD, looked more at the arm than the mouth (*p*_TD_ = 0.86, d_TD_ = 0.01; *p*_DS_ = 0.039, d_DS_ = 0.88). Finally, both groups looked longer at the eyes than the mouth (*p*_TD_= 0.001, d_TD_ = 0.4; *p*_DS_ = 0.035, d_DS_ = 0.89). To further understand this complex looking pattern across the groups we reduced the number of AOIs, thus we compared the face (including the eyes and the mouth), the arm, and the background separately for each group. We observed that both groups looked more at the background than the arm (*p*_TD_ = 0.01 d_TD_ = 0.32; *p*_DS_ = 0.0017, d_DS_ = 1.25) and more at the face than the arm (*p*_TD_ < 0.001, d_TD_ = 1.02; *p*_DS_ = 0.0017, d_DS_ = 1.22). However, only TD, but not DS infants, looked more at the face than the background (*p*_TD_ < 0.001, d_TD_ = 0.71; *p*_DS_ = 0.6, d_DS_ = 0.18). To directly compare the two groups, we computed a linear-mixed analysis on proportion of looking time with AOI (face, arm, background) and group (TD and DS) as fixed factors (with the interaction term), and by-subject as a random intercept. This analysis confirmed that the AOIs differed (F = 26.94, *p* < 0.001, η^2^_p_ = 0.38, 95% CI = 0.22–0.51), and more importantly that there was an interaction between AOI and Group (F = 3.21, *p* = 0.04, η^2^_p_ = 0.07, 95% CI = 0.01–0.18). Further pairwise comparisons (Bonferroni controlled) revealed that groups did not differ in their looking time to the arm (t = −0.03, *p* = 0.97, d = 0.06). However, results suggest a trend of TD looking more to the face than DS (MTD = 0.65, MDS = 0.48, t = 1.8, *p* = 0.074, d = 0.38), and a trend for TD looking less to the background than DS (MTD = 0.26, MDS = 0.42, t = −1.77, *p* = 0.079, d = 0.38).

Finally, considering that DS infants were slower in the visual orientation task, we tested whether individual latency in the visual orientation task modulated performance in the audiovisual task. To the previous mixed model analysis, we added the average latency for each subject as a fixed effect, while other parameters maintained the same. The results were similar as in the previous analysis, with a main effect of AOI (F = 26.64, *p* < 0.001, η^2^_p_ = 0.38, 95% CI = 0.22–0.51) and an interaction between AOI and Group (F = 3.17, *p* = 0.04, η^2^_p_ = 0.03, 95% CI = 0.01–0.18). The pairwise comparisons revealed the same pattern: TD and DS do not differ in their proportional looks to the arm (t = −0.02, *p* = 0.97, d= 0.006), while we observed a trend of TD looking more to the face than DS (t = 1.7, *p* = 0.09, d = 0.37), and TD looking less to the background than DS (t = −1.68, *p* = 0.09, d = 0.36). This suggests that even when average latency in the visual orientation task is taken into account the same pattern of findings across groups holds in the audiovisual task.

### 3.3. Correlation between Audiovisual Task and Communicative Skills

**Figure 4:**
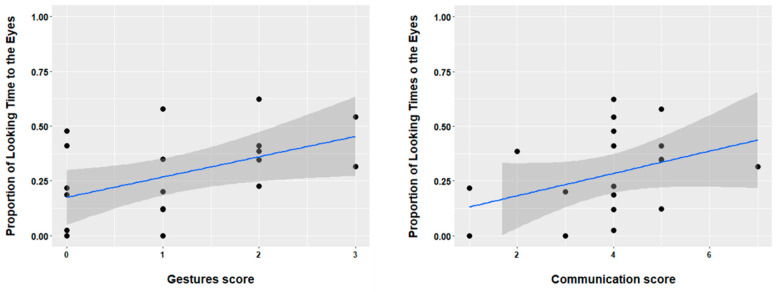
Scatter plot representing the relation between attention to the eyes in the audiovisual task, and gesture and communication score from the CSBS-DP for the TD group. Dots represent individual scores.

Using the CSBS-DP we correlated concurrent communicative skills with infants’ performance in the audiovisual task. For the current study we focused on skills that are relevant for infants’ performance in audiovisual communication. Therefore, we analyzed data (raw scores) from the following scales: emotion and eye gaze, communication, and gesture. Nineteen TD infants (mean age 6 months, range 6–6 months), and six DS infants (mean age 6.8, age range 6–8 months) provided CSBS data. Note that for those infants that were younger than 6 months (i.e., the minimum assessment age for the CSBS questionnaire) at the moment of the AV task, the CSBS data were collected later (i.e., within the time span of 2–3 months for 2 of the infants). We observed that in the TD group there was a significant positive correlation between the proportion of looking to the eyes in the audiovisual task and the score on the gesture scale (r(18) = 0.47, *p* = 0.01), as well the communication score (r(18) = 0.36, *p* = 0.05). These results are depicted in Figure 4. In addition, we observed a marginal correlation between looking to the arm and the gesture score (r(18) = 0.34, *p* = 0.06). Other areas of interest did not provide a significant correlation with the CSBS scales (all *p*s > 0.1). Finally, for the DS group we observed no significant correlations (all *p*s > 0.2).

## 4. Discussion

The current paper focuses on early abilities that might be supporting language development in DS and TD 5–7-months old infants. In particular, we assessed infants’ early visual attention, audiovisual speech processing, and communication skills. We will discuss each of the assessed measures and their implications for language development, particularly for DS infants. First, we observed that DS infants are slower in orienting their visual attention to stimuli in comparison to TD peers. This means that DS infants need more time to start attending to salient visual cues in their environment, here a flashing red light. This result is in line with previous studies on impaired visual attention in DS toddlers and children, especially in disengaging their visual attention [34,35,36]. However, our results differ from Landry and Bryson’s study [35] where DS preschool children were similar in the visual orientation task to TD children matched in mental age with the DS group. There are at least two explanations for these between-studies differences. First, we tested a much younger population than in Landry and Bryson [35] and it is possible that by the preschool age DS children do improve their visual orientation attention. Second, there are important methodological differences between studies. Note that we compared DS and TD infants that were matched in their chronological, rather than in their mental age. Considering that we were interests in assessing DS infants between 5–7 months of age it would be difficult, if not impossible, to match groups in their mental age. Moreover, our task required infants to turn and orient their head to lateral/central position to flashing lights, whereas Landry and Bryson [35] used a set up with a central monitor and two lateral monitors placed in front of the child. It is therefore possible that our task was particularly challenging for the Down syndrome group. Nevertheless, our study is one of the first studies demonstrating that in first half of the first year of life, DS infants are impaired in orienting their visual attention to salient stimuli.

The second component we assessed was infants’ attentional pattern during audiovisual speech processing. We presented an animated character that waived and talked at the infant. We were particularly interested in examining what visual speech/communicative cues infants attend to at this age early age. We observed that the two groups demonstrated certain similarities in their looking pattern: both groups do attend less to the waving arm at this age than the face and the background, suggesting that at this age the waiving gesture is not a particular salient communicative cue. Further, when attending to the face, both groups look more at the eyes than the mouth, in line with many recent studies done at similar ages (e.g., [41,42,43]). However, we observed a striking difference between the groups: TD infants attend more to the face than to the background, whereas DS infants attend similarly (~40 percent of their looking time to the screen) to the background and the face. This suggests that for DS infants the face is not a salient cue, at least not more salient than the background. Further implying that when observing a scene, DS infants attend equally to social/speech cues and to other cues that are not relevant for communication. This finding is in line with other studies that observed impairment in face and audiovisual speech processing in DS infants and toddlers [31,32,33]. Considering that we observed that DS infants are also slower to orient their visual attention, we can propose that such impairment affects their ability to orient to and fixate salient audiovisual speech cues (e.g., the face). Therefore, early interventions that are based on improving communication abilities have to take into account that DS attention to communicative cues is impaired. Moreover, we detected such impairment already in the first half of the first year of life, suggesting that interventions could target the ability to detect visual speech cues, even before interventions focusing on improving joint attention take place. We could also speculate that improving visual orientation might improve their ability to detect visual speech/communication cues and further research should address this possibility. It is important to note, however, that we have assessed audiovisual speech processing using an animated character, that certainly differs from a human face. In particular, the richness of the interplay between the acoustic signal, articulatory movements and facial expressions is reduced in animated characters, in comparison to a human face. Interestingly, even with the animated character, DS and TD infants attend more to the eyes than the mouth, similar to previous studies assessing attention to a human face (e.g., [41,42,43]). Moreover, we also found that DS infants differ from TD infants in how they attend to communicative cues in an animated character talking face. It might be possible that for DS infants a human talking face would elicit greater attention, and future research should assess DS attention to a talking human face in relation to attractive background.

Finally, we observed important across-group differences regarding the relation between attending to audiovisual communicative cues and communicative development. First, we observed that attention to the eyes in the TD group relates to concurrent gesture and communication skills (and a trend for a positive correlation between looking to the arm and gesture skills was also found). Note that the items in the gesture and communication scales are mostly tackling non-verbal communication skills. For instance, assessing whether the child is pointing, waiving, asking for attention when a caregiver is not providing it, etc. So, we observed that in ~6-month-old typically developing infants, attending to relevant audiovisual cues, particularly to the eyes and the arm, supports early communication skills. This result is in line with previous studies on the importance of audiovisual cues for communication skills in typical development (e.g., [44]). However, in DS infants, we have not observed patterns supporting a relationship between performance in the audiovisual task and communication skills. We further inspected these results and observed that all DS infants have value 0 on the gesture score, meaning that gesturing in 5–7-month-old DS infants did not emerge yet. Considering that we have observed that DS infants attend much less to relevant audiovisual cues than TD infants, it is possible that less attention to audiovisual cues hinders their communicative skills. Alternatively, their poor communicative skills could drive away their attention from relevant audiovisual speech/communication cues. Either way, we observed that in the first 6 months of life, DS infants’ link between audiovisual communicative attention and communication skills is not yet established. Further research is needed to address what is the relation between early attention to communicative cues and later communicative development.

A limitation of our study is certainly the small sample size, and future work should include larger samples. However, it should be noted that the population of DS infants is far less than that TD infants. According to the report from the National health institute [45] in the period from 2008–2017, in average, ~20 Down syndrome infants were born in Portugal per year. Thus, we assessed 35% of the population in a given year. An additional limitation of the current study is that our DS group was chronologically age matched with the TD group, and not mental age matched. Therefore, it is possible that the findings might change if groups were matched by mental age, in particular if an older group of DS infants was considered instead. However, that would leave our goal of investigating very early attention and audiovisual abilities in DS infants unaddressed, as well as of contributing to understand how DS communicative abilities develop from an early age. In future work, we plan to look at older DS infants to examine how these abilities develop. Nevertheless, based on the current findings we could speculate that undeveloped attentional orientation skills hinder DS infants’ orientation to speech/communication cues. Therefore, future research should also explore intervention strategies that would focus on improving orientating attention, but specifically to audiovisual speech/communication cues, i.e., the face.

## 5. Conclusions

The current study assessed early attentional and audiovisual processing abilities in typically developing and in Down syndrome infants at 5–7 months of age. The study showed, for the first time, that at such an early age DS infants’ attention and audiovisual speech processing is following a different developmental path than typically developing infants. We also observed that audiovisual attention supports concurrent communicative abilities in TD infants, but not in DS infants. In short, the current study demonstrated that early visual attention and audiovisual speech processing might be impaired in DS infants with consequences for their communication development, opening new avenues for early interventions in this clinical population. Furthermore, results from this study suggest that in face-to-face communication, DS infants might need more time to detect/attend to communicative cues, and caregivers might emphasize from early age face-to-face communication as a form of training attention to communicative cues.

## Data Availability

The raw data supporting the conclusions of this article will be made available by the authors, without undue reservation, on request to the corresponding author.
